# Design and Results of a Culturally Tailored Cancer Outreach Project by and for Micronesian Women

**DOI:** 10.5888/pcd9.100262

**Published:** 2012-04-05

**Authors:** Nia Aitaoto, Kathryn L. Braun, Julia Estrella, Aritae Epeluk, JoAnn Tsark

**Affiliations:** Papa Ola Lokahi. Ms Aitaoto is also affiliated with the University of Iowa; ‘Imi Hale-Native Hawaiian Cancer Network U01CA114630, Papa Ola Lokahi, Honolulu, Hawai‘i, and University of Hawai‘i, Office of Public Health Studies, John A. Burns School of Medicine, Honolulu, Hawai‘i; Micronesians United, Honolulu, Hawai‘i; Micronesians United, Honolulu, Hawai‘i; ‘Imi Hale-Native Hawaiian Cancer Network, Papa Ola Lokahi, Honolulu, Hawai‘i

## Abstract

**Background:**

In 2005, approximately 26% of Micronesian women aged 40 or older in Hawai‘i used mammography for breast cancer screening. We describe an 18-month project to increase screening participation in this population by tailoring educational materials and using a lay educator approach.

**Community Context:**

New immigrants to Hawai‘i are Marshallese from the Republic of the Marshall Islands and Chuukese, Pohnpeians, and Kosraeans from the Federated States of Micronesia. In Hawai‘i, these 4 groups refer to themselves collectively as Micronesians, although each group has its own distinct culture and language.

**Methods:**

From 2006 through 2007, we applied principles of community-based participatory research —trust building, joint assessment, cultural tailoring of materials, and skills transfer —to develop and track the reach of Micronesian women lay educators in implementing a cancer awareness program among Micronesian women living in Hawai‘i.

**Outcome:**

Using our tailored in-language materials,  11 lay educators (5 Chuukese, 3 Marshallese, 2 Pohnpeians, and 1 Kosraean) provided one-on-one and small group in-language cancer information sessions to 567 Micronesian women (aged 18-75 years). Among the 202 women aged 40 or older eligible for mammography screening, 166 (82%) had never had a mammogram and were assisted to screening appointments. After 6 months, 146 (88%) of the 166 had received a mammogram, increasing compliance from 18% to 90%. Lay educators reported increases in their skills and their self-esteem and want to extend their skills to other health issues, including diabetes management and immunization.

**Interpretation:**

Tailoring materials and using the lay educator model successfully increased participation in breast cancer screening. This model may work in other communities that aim to reduce disparities in access to cancer screening.

## Background

New immigrants to Hawai‘i include Pacific Islanders from the US-associated Pacific Islands (USAPI) (1). No population-based studies of breast cancer prevalence or screening in these populations have been conducted either in their home countries or in the United States. However, program-level data suggest low participation in breast cancer screening. A 2003 assessment of the 6 USAPI jurisdictions found mammography units on only 2 of the more than 500 inhabited islands of the Republic of the Marshall Islands (RMI) and on only 1 island of the 132 inhabited islands of the Federated States of Micronesia (FSM) (2). A chart review conducted by a community health center in Hawai‘i frequented by Micronesians found that only 26% of female Micronesian patients aged 40 or older, the age at which yearly mammograms are recommended to begin, had ever had a mammogram (3,4). In subsequent focus groups, Micronesian women identified several barriers to breast cancer screenings: 1) low awareness of screening, 2) lack of Micronesian-language educational materials, and 3) no health insurance or limited financial resources for copayments. A resource for migrant women is the Breast and Cervical Cancer Control Program (BCCCP), which provides free breast and cervical cancer screening to uninsured and underinsured women. However, data from the Hawai‘i BCCCP suggest that few Pacific Islanders use this service (5). The purpose of this article is to describe an 18-month project designed in partnership with Micronesian women to increase breast cancer screening in this population.

## Community Context

In 2000, an estimated 12,000 to 15,000 people from the USAPI were living in Hawai‘i (6). The USAPI refers to 6 separate and distinct island-based jurisdictions in the Pacific that have formal relationships with the US government: American Samoa, Guam, the Commonwealth of the Northern Mariana Islands, FSM (inclusive of the 4 states of Chuuk, Kosrae, Pohnpei, and Yap), Palau, and RMI. Each jurisdiction has a distinct culture and language, but they share a history of colonization. All 6 jurisdictions have been used by the US government for military purposes, including nuclear weapons testing, which increases cancer risk. Today, all 6 jurisdictions have strong economic and political relationships with the United States and use the US dollar as currency. By formal agreement, USAPI residents can migrate freely to the United States. Many do so in pursuit of education, work, and health care opportunities, which are limited in their home countries (2,6-8). The largest USAPI migrant groups in Hawai‘i are Marshallese from RMI and Chuukese, Pohnpeians, and Kosraeans from FSM. In Hawai‘i, these 4 groups refer to themselves collectively as Micronesians.

Two organizations collaborated on this project. ‘Imi Hale Native Hawaiian Cancer Network (www.imihale.org) is 1 of 23 Community Networks Program Centers funded by the National Cancer Institute's Center to Reduce Cancer Health Disparities. Founded in 2000, ‘Imi Hale's work is guided by principles of community-based participatory research (CBPR), emphasizing community involvement, capacity building, respect for cultural values, and information sharing (9,10). Micronesians United (MU) is a grassroots organization formed in 1999 to unite people from RMI and FSM to work together for the betterment of their families in Hawai‘i. We worked together to develop and implement the program, using CBPR methods (Table 1). We outline the project's critical components, summarize evaluation findings, and conclude by describing lessons learned.

## Methods

Adhering to CBPR principles, the development process included 4 steps: 1) building relationships and identifying champions; 2) working together to assess needs, barriers, and desired solutions; 3) culturally tailoring materials and tools; and 4) training lay educators. At each step, we supported the transfer of knowledge between the 2 organizations and the empowerment of MU members and Micronesian women to address this and other issues (10). This project was approved by the institutional review board of the Native Hawaiian Health Care Systems in Honolulu.

### Building relationships and identifying champions

In 2001, ‘Imi Hale began working with MU, providing the organization pro bono assistance with grant writing. ‘Imi Hale also provided advice on community mobilization, organizational development, and health-related workshops; participated in MU health fairs; and paid for the translation and printing of in-language brochures. Therefore, a trusting relationship had been established between the 2 organizations by 2005, when MU asked for help to increase breast cancer awareness and screening in its community. Several meetings were held in the community, and by group consensus we identified a champion in each organization to lead project development. These champions had many years of experience in advocating for, coordinating, and implementing health projects for Pacific Islanders.

### Joint assessment

To gather information about the problem and discuss potential solutions, the team conducted a focus group in October 2005 with 16 women aged 22 to 69 years who were affiliated with MU and who represented the 4 cultural groups. Using methods that worked in the Samoan community in Hawai‘i (11), we started each session with a prayer, served food, and allowed the group to determine the amount of time spent on each question. The focus group was conducted in English, with assistance of translators for the 4 languages. Questions were: 1) What are your leading health concerns? 2) What are the barriers to good health? and 3) What cultural strengths can help improve the health of Micronesians in Hawai‘i? Findings were recorded in English on posted newsprint.

Fifteen of 16 focus group participants identified breast cancer as a leading concern. Awareness about breast cancer was high because of recent diagnoses among the membership. Participants then identified characteristics of Micronesian culture in Hawai‘i, including facilitators and barriers to breast cancer screening, both of which would need to be considered when developing our program (Table 2). Most participants knew that the United States tested nuclear weapons on their islands (2) but were not clear about the relationship between cancer and radiation from nuclear testing. They knew of no educational materials in their languages.

Participants discussed the limited health care resources in Micronesia. Because of limited resources, many cancers in Micronesia are diagnosed late and cause death. Therefore, many Micronesians are fatalistic about cancer. Only half knew about the breast and cervical cancer screening programs funded by the Centers for Disease Control and Prevention in their home countries or in Hawai‘i. Those that knew about the Hawai‘i BCCCP said it did not extend to Micronesian communities in Hawai‘i. Participants also said that local providers were unfamiliar with Micronesian cultures and health-seeking behaviors. We learned from participants that Micronesians are shy and find discussions about "private parts" to be offensive; they felt that health care providers should provide "apologies" for using "offensive" language. Women who wanted to have a mammogram felt hesitant to make appointments by telephone and anticipated difficulties arranging transportation, taking time for appointments, and paying for services.

In terms of strengths, participants discussed the collectivistic orientation of Pacific Islander cultures and the expectation of reciprocity. Many Micronesians belong to family, church, and community groups and can be reached through these venues. Women usually congregate separately from men, and women usually help each other with child care and other tasks and give each other advice about health and daily living.

Therefore, focus group participants recommended that MU members be educated about breast cancer and serve as one-on-one peer educators and navigators of Micronesian women in the community. Given the group and family orientation of these cultures, women recommended that breast cancer education be provided to women of all ages. Participants recommended that women aged 40 or older learn about and undergo mammography and that younger women support older women and learn about their own need for routine clinical breast exams.

The champions reviewed literature about lay educator cancer programs to help us think about what we needed for a cancer-awareness program for Micronesian women (12-15). Objectives for the 18-month project were to 1) culturally tailor a training curriculum and toolkit for Micronesian lay educators, 2) translate materials into 4 Micronesian languages (Marshallese, Pohnpeian, Chuukese, and Kosraean), 3) recruit and train a minimum of 12 women as lay educators, and 4) track the effect of the lay education program (ie, number of women reached and referred to mammography).

### Culturally tailoring materials and project tools (July-December 2006)

The first step in tailoring education materials was finding and reviewing existing breast cancer education materials and tools used by lay educators in other communities. The group selected a number of pieces that they felt were appropriate and adaptable for Micronesian women, including a portable flip chart developed for the lay education project of the Deep South Network for Cancer Control, breast education brochures and shower hangers developed by ‘Imi Hale, and 2 tactile breast cancer education pieces — a beaded key chain distributed by the American Cancer Society Hawai‘i Chapter and a multiple-sized beaded necklace produced by ‘Imi Hale. We developed a resource information card specific to this project with referral numbers of the BCCCP providers and other breast cancer screening providers. We also developed carbon-copy referral slips and a journal for lay educators to record information, questions, and concerns that could be discussed at their weekly meetings. Although the project focused on breast cancer screening, basic information on cervical cancer screening was included in the flip chart. Women of all ages were encouraged to have a regular Papanicolaou test (Pap smear), although their participation in cervical cancer screening was not systematically tracked in this project.

The next steps were tailoring and translating the materials for relevance and appropriateness to Micronesian women on the basis of the information received in the focus group. For example, we used Micronesian images in our materials, which distinguished them from mainstream materials and signaled that the project leaders had taken the time to create materials especially for Micronesians (http://www.imihale.org/education_materials.htm). The educational materials were translated into the 4 Micronesian languages by the leaders of MU. Translations were not verbatim from English to the target languages but instead used traditional metaphors, allegories, and proverbs to help explain difficult medical and scientific concepts in ways that were acceptable and understandable (16).

Once finalized, the toolkit materials were pilot tested with 80 Micronesian women (MU members; 20 women for each of the 4 languages), who inspected and discussed the pieces during focus groups and in one-on-one meetings led by native speakers. These women gave valuable suggestions for improving the look and wording of the materials. Women appreciated the inclusion of Pacific images, which they said made the materials "touch our hearts and inspire us to action."

### Transferring skills (January-March 2007)

We recruited 16 Micronesian women to be trained as navigators. All were elders or respected community members and spoke at least 1 of the 4 target languages. All expressed interest in helping their communities and a willingness to complete the training. The 6-hour training curriculum, which was developed based on the toolkit and existing ‘Imi Hale materials, was provided in four 90-minute sessions over 4 weeks. Navigators learned about breast cancer, Micronesian women's high-risk status resulting from nuclear weapons testing in their home islands, and health care resources. Lay educators heard from cancer survivors to help counter the belief that cancer equates with death. They visited mammography clinics to see the machines and meet the staff who conducted the screening. Professional behavior was discussed, including the need to attend training, how to make phone calls and referrals, and how to document peer contacts. Also discussed was the Western notion of time versus Pacific Islander time, which is much more flexible and subjective, and the importance of honoring Western time when making and keeping mammography appointments. Once trained, navigators received a name badge and business cards to formalize their roles. They also received their toolkit in a distinctive colorful bag that was both culturally appropriate and practical.

During the next 3 months, lay navigators provided cancer educational presentations to Micronesian women, using the materials tailored to this population. In line with the cultural value of reciprocity, each woman who attended an education session was given a bead necklace kit and bead keychain. These gifts were not only attractive but also educational, because they demonstrated the sizes of cancerous lumps found through mammography and through clinical breast examination and breast self-examination. Women aged 40 or older who needed a mammogram were assisted in making appointments for and directed to screening. Women who were not accompanied to screening showed their mammography results to the lay educator as proof of screening. The educators asked the women about their screening status after the education session and recorded data on a spreadsheet.

Lay educators agreed to meet weekly during the outreach phase. Meetings lasted 2 hours and were facilitated by ‘Imi Hale and MU champions. In meetings, lay educators described how they conducted one-on-one and group breast cancer education sessions, provided referral information, and made screening appointments. They compared notes on their successes and challenges, and they sought answers to emerging questions about education and navigation. Grant funding was available to pay each lay educator a stipend of $100 per month ($300 total) to offset costs associated with outreach. Many women used the stipend toward transportation for themselves and the women they educated and navigated.

## Outcome

Of the 16 lay educators who were trained, 5 dropped out early in the project, including 2 who moved back to their home countries and 3 who were too busy. The mean age of the remaining 11 lay educators was 55 years. Five were Chuukese, 3 were Marshallese, 2 were Pohnpeian, and 1 was Kosraean. Six were pastors' wives, 2 were teachers, 2 were translators, and 1 was a retired nurse; 2 were breast cancer survivors. Four had attended vocational school, 5 had 2-year college degrees, and the other 2 had some college. All had lived in the United States from 4 to 11 years (mean, 7.1 y).

In their efforts to reach 20 women per month, lay educators first talked to their relatives, friends, and neighbors; once they were comfortable with their presentations, they extended their outreach to churches, Micronesian neighborhoods, homeless shelters, and other places where Micronesian women gather. Although they each hoped to reach 60 women in 3 months, they each educated between 48 to 54 women (mean, 51 women), for a total of 567 Micronesian women aged 18 to 75.

Of the 567 women educated, 324 were under age 40. Most (95%) had children, so they knew about and had participated in cervical cancer screening during pregnancy. They were taught about the importance of the clinical breast exam and of encouraging older women to get mammograms. Among the 202 women aged 40 or older and eligible for mammography screening, 166 (82%) had never had a mammogram and were assisted to screening appointments. Those women linked to a BCCCP provider for a mammogram also participated in cervical cancer screening, if needed. After 6 months, 146 (88%) of the women who had never had a mammogram had received one. Therefore, the percentage compliant with mammography screening recommendations increased from 18% (36/202) to 90% (182/202). An interesting practice that evolved from the program was the "group mammogram," by which lay educators arranged back-to-back mammogram appointments for 5 to 10 women at a time. All women went together to the clinic and stayed until the last woman was screened.

During the 3-month outreach and 6-month follow-up phases, 10 breast cancers were diagnosed. All of the women with breast cancer either had insurance or were screened through BCCCP, so their treatment was covered. Unfortunately, 2 women were diagnosed with late-stage breast cancer. Although hearing this news was difficult for the group, it underscored the importance of their job as educators and their need to get women engaged in regular screening so that cancers could be caught early, when they are most curable.

We noticed increased self-esteem, confidence, and capacity among the lay educators; they were able to keep their outreach schedules, present the information with confidence, arrange mammogram appointments, and accompany women to the clinics. During weekly meetings, they expressed how thrilled they were to be useful in their communities in Hawai‘i. In Micronesia, women traditionally serve as advisors and health promoters, but heretofore Micronesian women in Hawai‘i have been afforded few opportunities to do so. They also mentioned that it felt good to do something positive to counteract the negative images of Micronesians in Hawai‘i (17,18).

## Interpretation

The lay educator approach to increasing health awareness and preventive health practices is not new. Since our program, many publications have presented lay cancer education programs in different parts of the United States, focusing on various populations (19-21). As other investigators have found, lay navigators are effective in transferring information about cancer screening and survivability to underserved communities.

We confirmed that the CBPR process was useful in developing lay navigator training and materials for this unique community (10,16). Following CBPR principles, we worked together to assess needs and strengths and paid attention to cultural values to ensure project fit. The lay educator training was respectful of Pacific customs, processes, and practices. Involving women with a range of ages in education sessions and scheduling back-to-back mammogram appointments for a group of women fit well with Micronesian values of collectivity and mutual support. The Pacific manner of reciprocity and gifting also was respected. Lay educators expressed pride in their new but traditionally expected roles as caretakers and healers. Their desire to extend this model to address other health concerns, such as diabetes management and immunizations, demonstrates the acceptability and applicability of this process in the Micronesian community.

Without champions, projects such as ours are not likely to progress (22). Both partners had identified champions who assumed leadership roles and encouraged action among community members. ‘Imi Hale's champion had a long-standing relationship with the MU group and is fluent in several Pacific languages. The MU champion was a founding member of the group who was a trusted advocate for Micronesian issues. Their leadership of the weekly meetings ensured a safe forum for lay educators to discuss successes and concerns, solve problems, and translate their new knowledge and skills into practice with remarkable speed.

There were several limitations to implementing this project. First, project funding was limited, so our follow-up time was short. However, findings suggest that 88% of women who needed a mammogram received one. Second, we did not ask the women who interacted with the lay educators how they perceived the project; therefore, we do not know how the community received the lay educators or what really motivated them to access screening. Third, the lay educators did not meet their goal of 20 women per month because the outreach and education process took longer than expected. Finally, we were unable to follow up on the 10 women diagnosed with breast cancer. From our discussions, we understand that they were linked to treatment services after diagnosis, but we do not know how many completed treatment or what their treatment outcomes were.

Despite limitations, this project successfully demonstrated the appropriateness and success of the lay educator approach in promoting breast cancer screening in Micronesian communities. It also confirmed the effectiveness of using CBPR processes in the design and implementation of programs targeting minority and immigrant populations in the United States. This article can serve as a step-by-step guide for other groups that want to help special populations expand cancer screening.

## Figures and Tables

**Table 1.** Timeline for 18-Month Project to Develop and Begin Implementing Micronesia Lay Educator Program, Hawai‘i, 2006-2007

**Time Frame**	**Tasks**
Months 1-3 (April-June 2006)	Building on history of mutual trust, Micronesians United and ‘Imi Hale representatives Discuss ideas Identify champions from within the 2 agencies, Micronesians United and ‘Imi Hale Conduct focus groups Review literature
Months 4-9 (July-December 2006)	Champions work together to Design the program Assess existing resources Culturally tailor program materials and tools Train lay educators
Months 10-12 (January-March 2007)	Lay educators Provide education and outreach to female family members, neighbors, friends, and others Track encounters and outcomes Receive stipends Champions work together to organize and provide continuing education
Months 13-18 (April-September 2007)	Lay educators Continue to link women to screening Continue to track encounters and outcomes Think about next steps Champions Analyze data and write reports Work together to help lay educators realize their next steps

**Table 2:** Barriers and Strengths Identified by Micronesian Women and How They Were Considered in Program Development, Hawai‘i, 2006-2007

**Factors**	**Micronesian Characteristics**	**How Incorporated Into the Lay Educator Program**
Historical and geographic	Knowledge of the history of nuclear testing in the region, but unclear on links between radiation and cancer Scarcity of cancer detection, staging, and treatment services in region, leading to high rates of late-stage diagnosis and death	Educated women about risks of radiation exposure Educated women about services available in Hawai‘i, the expectation for US women to get screening, and the benefits of early diagnosis and treatment
Immigrant status	Unfamiliar with US health services, especially cancer-care services Unfamiliar with US health-seeking norms (eg, need for making and keeping appointments) Unfamiliar with using phones and/or lack of personal telephone Low household income, limited options for transportation and health insurance Lack of extended family network in United States (eg, to help with childcare, transportation, support) English is not first language Few Micronesians and Micronesian language interpreters in US health care workforce; existing providers seem insensitive Existing education materials not relevant Discrimination against Micronesians who are seen as "takers" rather than "contributors" to society	Trained lay educators in cancer-care services by taking them on facility tours and providing opportunities to meet providers Trained lay educators about importance of making and keeping appointments, including a serious discussion of "Western" vs "Pacific" time Trained lay educators in using telephone to make appointments and to contact women about keeping appointments Lay educators linked women to BCCCP Lay educators learned all public transportation routes and used stipend to help get women to screening appointments Lay educators helped arrange childcare and transportation and provided support Translated education materials into 4 Micronesian languages Lay educators became another level of provider, giving basic information in Micronesian languages and linking women to cancer screening and treatment Cultural values and images were incorporated into education materials Lay educators noted that program helped them contribute to the health and well-being of their community, countering stereotypes
Cultural	Culturally inappropriate to discuss "private parts," especially without sufficient "apology." High levels of modesty Shyness Belief that cancer is God's will and that cancer equates to death Women expected to focus on the home and family Collectivistic orientation, rather than individualistic Traditional roles of women as caretakers and healers for extended family and group Reciprocity is important; civic engagement includes giving and receiving	Attention paid to reducing use of "offensive" words and apology provided and forgiveness requested before discussing private matters; lay educators accompanied women to exams and helped to find and use examining robes Program designed so that lay educators would be one-on-one, rather than group, educators Provided basic information on cancer and early detection and arranged for women to hear stories from cancer survivors Lay educators helped provide or arrange others to provide childcare so that women could get screened Organized training activities in groups Lay educators arranged back-to-back appointments for women so that a group could go together to screening Program offered women a chance to fill traditional roles as caretakers and healers Lay educators received toolkit and badge and gave gifts (eg, beaded necklace, keychain)
